# The Colony Stimulating Factor-1 Receptor (CSF-1R)-Mediated Regulation of Microglia/Macrophages as a Target for Neurological Disorders (Glioma, Stroke)

**DOI:** 10.3389/fimmu.2021.787307

**Published:** 2021-12-07

**Authors:** Cristina Barca, Claudia Foray, Sven Hermann, Ulrich Herrlinger, Isabel Remory, Damya Laoui, Michael Schäfers, Oliver M. Grauer, Bastian Zinnhardt, Andreas H. Jacobs

**Affiliations:** ^1^ European Institute for Molecular Imaging (EIMI), University of Münster, Münster, Germany; ^2^ Division of Clinical Neuro-Oncology, Department of Neurology, University Hospital Bonn, Bonn, Germany; ^3^ Centre of Integrated Oncology, University Hospital Bonn, Bonn, Germany; ^4^ In vivo Cellular and Molecular Imaging laboratory (ICMI), Vrije Universiteit Brussel (VUB), Brussels, Belgium; ^5^ Myeloid Cell Immunology Lab, VIB Center for Inflammation Research, Brussels, Belgium; ^6^ Lab of Cellular and Molecular Immunology, Vrije Universiteit Brussel, Brussels, Belgium; ^7^ Department of Nuclear Medicine, University Hospital Münster, Münster, Germany; ^8^ Department of Neurology with Institute of Translational Neurology, University Hospital Münster, Münster, Germany; ^9^ Biomarkers & Translational Technologies (BTT), Pharma Research & Early Development (pRED), F. Hoffmann-La Roche Ltd., Basel, Switzerland; ^10^ Department of Geriatrics and Neurology, Johanniter Hospital, Bonn, Germany

**Keywords:** colony stimulating factor-1 receptor, neuroinflammation, glioma, stroke, microglia/macrophages, positron emission tomography

## Abstract

Immunomodulatory therapies have fueled interest in targeting microglial cells as part of the innate immune response after infection or injury. In this context, the colony-stimulating factor 1 (CSF-1) and its receptor (CSF-1R) have gained attention in various neurological conditions to deplete and reprogram the microglia/macrophages compartment. Published data in physiological conditions support the use of small-molecule inhibitors to study microglia/macrophages dynamics under inflammatory conditions and as a therapeutic strategy in pathologies where those cells support disease progression. However, preclinical and clinical data highlighted that the complexity of the spatiotemporal inflammatory response could limit their efficiency due to compensatory mechanisms, ultimately leading to therapy resistance. We review the current state-of-art in the field of CSF-1R inhibition in glioma and stroke and provide an overview of the fundamentals, ongoing research, potential developments of this promising therapeutic strategy and further application toward molecular imaging.

## Introduction

Inflammation is a biological process triggered by injuries, infections and damages suffered by the cells that disrupt tissue homeostasis. Together with the innate and adaptive immune responses, they are discussed as essential factors in the onset and progression of many neurological conditions (1). Therefore, the use of neuroprotective and immunomodulatory agents that curtail inflammation has become an essential area of research. The failure of clinically effective translation is partly due to the complexity of molecular alterations and the spatiotemporal functional dynamics of the different cellular players. Still, targeting specific inflammatory and immune pathways represents a promising therapeutic strategy in many neurological diseases requiring further investigations (1). One of the major players highly investigated is microglia (2–5). As part of the resident immune cells, microglia quickly activate after injury by producing chemokines, cytokines and other signalling molecules. These cells show evolving detrimental pro- and/or beneficial anti-inflammatory properties, worsening and/or promoting tissue repair, respectively (6, 7). Their spatiotemporal function and contribution to disease have been extensively investigated using different systems (genetic animal models, drug-based interventions) (8, 9). In this review, we focus on the pharmacological intervention employing the colony stimulating factor-1 receptor (CSF-1R) inhibitors, which stands as a powerful drug-based approach to study microglia dynamics under inflammatory conditions, with a promising translational value (10).

## Microglia

Microglial cells serve as regulators of homeostasis in the central nervous system and represent the first line of defence against infection and injury (11). They are long-living cells and have an intrinsic capacity for self-renewal (12, 13). Highly ramified, they continuously sense the local environment by extending and retracting their processes (14, 15). In case of injury or infection, microglia are highly dynamic cells capable of undergoing quick transcriptome changes depending on the type of signals sensed in their environment (15).

They play a significant role in neuronal plasticity and synaptic connections (14, 16). They shape neuronal networks and control synaptic pruning, serving an essential role in learning and memory. Furthermore, microglia secrete neurotrophic factors that affect synaptic plasticity and promote synapse formation, including insulin-like growth factor 1 (IGF-1), brain-derived neurotrophic factor (BDNF) and transforming growth factor (TGF)-β (17). Mice depleted of microglia showed deficits in learning tasks and significantly reduced synapse formation (16). It highlights the importance of microglia in activity-dependent plasticity, with proper neuron-microglia cross-talk essential for neural network landscape (18).

Microglia show region-dependent molecular and transcriptional heterogeneity in physiological conditions. Masuda et al. (19) reported the existence of ten microglia subtypes in the healthy mouse brain (19, 20). Ten clusters (C1-C10) were differentiated by their different gene expression profile, including C1-C6 to be embryonic microglia and C7-C10 to be postnatal microglia. Results indicated that *tmem119*, *Selplg* and *Slc2a5* markers were highly expressed in postnatal microglia compared to embryonic microglia, indicative of cell maturation. It was suggested that the four postnatal clusters might be related to different cell functions (19). Additional clusters were observed in inflammatory conditions, such as demyelinating and neurodegenerative diseases, suggesting that a pathological environment can trigger additional disease-specific microglial subpopulations (20, 21), displaying enriched disease-related genes. Moreover, some microglia clusters may also be depleted in neurodegenerative diseases (22).

A major limitation in tracking microglia-specific contributions to different pathological pathways is that they share many common features with bone marrow (BM)-derived macrophages, including morphologies, surface markers and other characteristics (23). That explains the frequent use of the terminology microglia/macrophages to describe this family of mononuclear myeloid cells. In response to infection or injury, peripheral myeloid cells (including macrophages, bone marrow-derived monocytes, etc.) are recruited to the injured tissue and exhibit similar morphology and expression patterns to microglia, forming a pool of indistinguishable activated myeloid cells (24). However, microglial cells do have a unique transcriptomic signature, and therefore they potentially exert different functions compared to macrophages. Microglia also show physiological differences: (i) resident microglia cells are long-lasting cells, (ii) they self-renew and (iii) they are not replaced by peripheral bone marrow-derived cells (17). Recently, Butovsky et al. described putative 89 markers for resident microglial cells (5), including *P2ry12*, *Tmem119*, *Olfml3*, *Hexb*, *Sall1*, etc., identified in gene-expression studies. Different genetic and pharmacological strategies have been implemented and are currently developed to investigate microglia/macrophages functions, including genetic ablation or inhibition of the previously reported markers. A promising approach includes inhibiting the colony stimulating factor-1 receptor by small molecule inhibitors since this receptor is almost exclusively expressed by microglial cells in a steady-state brain where it regulates their developmental functions, including survival, differentiation, and proliferation.

## CSF-1R Inhibition-Induced Depletion and Repopulation in Physiological Conditions

The colony-stimulating factor-1 receptor (CSF-1R), also known as macrophage colony-stimulating factor (M-CSF) receptor, is a transmembrane tyrosine kinase receptor found at the cell surface of microglial cells, bone-marrow-derived macrophages, monocytes, and other cell types (osteoclasts, dendritic cells). The CSF-1/CSF-1R axis regulates cell survival, proliferation, differentiation, and functions of the mononuclear phagocytes (25, 26).

CSF-1R exists as an autoinhibited form and activates through dimerization and auto-phosphorylation of several tyrosine residues initiating a signalling cascade and the internalization of the receptor. The cascade is activated upon binding the endogenous CSF-1 or interleukin-34 (IL-34) and includes PI3K-AKT and AMPK pathways implicated in macrophages differentiation. Both cytokines promotes macrophages survival, differentiation and proliferation but show different ability to polarize macrophages (27). They share low primary sequence homology, but show similar folding/tertiary structure and interact with overlapping regions of CSF-1R (26). They exhibit different spatiotemporal patterns of expression and play complementary roles during development and adulthood. In the brain, CSF-1 is primarily expressed by the innate immune cells (astrocytes, microglia and oligodendrocytes) while IL-34 is secreted by neurons. IL-34 acts locally, not only on CSF-1R, but also on protein tyrosine phosphatase-z (PTP-z) and CD138, while CSF-1 is also found in the circulation and selective for CSF-1R (26). Furthermore, blocking of CSF-1 and IL-34 led to significant depletion in white and grey matters respectively, highlighting that those cytokines are differentially required for microglia maintenance in the different brain compartments (28). Accordingly, microglia are reduced by 30% in Csf1-null brains while reduced by 70% in IL-34-null brains but almost fully depleted in CSF-1R^-/-^ deficient mice (29).

A low level of CSF-1 stimulates microglia survival and inhibits protein degradation, while increased CSF-1 expression, as observed in inflammatory conditions, was associated with cell proliferation and enhanced migration (26). Additionally, CSF-1R activates several regulators of multipotent progenitor cell differentiation, directing the cell fate toward monocyte/macrophages or granulocytes.

Many CSF-1R inhibitors have been developed (Dasatinib, PLX3397, PLX5622, Ki20227, PLX647, GW2580). Among them, PLX5622 (Plexxikon Inc.) is a potent inhibitor of the kinase activity (KI = 5.9 nM) showing high selectivity over other kinases (29, 30). X-ray crystal structure of the CSF-1R-PLX5622 complex shows that PLX5622 binds to the active site (pocket) of the CSF-1R by forming hydrogen bonds (30).

Under physiological conditions, CSF-1R inhibition causes a reversible depletion of the microglial population within a few days (29, 31). Roughly, around 50% of the microglia population is depleted within three days and over 90% after one week of treatment (29), with sustained effect over the month with continuous administration (29, 31). Elmore et al. (29) showed that depletion was induced *via* apoptosis (29) and did not result from the cell dedifferentiation into an intermediate cell type (32). Depletion did not have a discernible impact on baseline inflammation-related markers level (ROS, cytokines). However, investigations on the resistant Iba-1^+^ cells in wild-type brains indicated that those cells displayed elevated inflammatory chemokines and proliferation marker and reduced homeostatic markers expression (13).

Elimination of CSF-1R^+^ cells has no apparent long-lasting impact on neurological functions (29, 33, 34). Torres et al. (34) showed a transient alteration in spatial learning and memory after seven days of treatment (PLX3397) that vanished after 21 days, as previously reported by others (29). Additionally, no apparent effect on brain volume or blood-brain barrier integrity was observed under physiological conditions (29).

Besides, peripheral myeloid cells, including monocytes/macrophages, also express CSF-1R. Therefore, the depletion of microglia may affect the baseline peripheral immune response depending on the duration and way of administration. Otxoa-de-Amezaga et al. (35) observed a reduction in a minor subset of blood Ly6C^-^ monocytes which are dependent on CSF-1R (35). A recent investigation reported that systemic PLX5622 treatment leads to broad myelosuppression and has long-term consequences even after drug withdrawal (36). CSF-1R inhibition significantly reduces CCR2^+^ monocytes, F4/80^+^ and MerTK^+^ cells, T lymphocytes in bone marrow, and also spleen and blood cell populations (36).

PLX5622 treatment has minimal impact on neurons while its effect on astrocytes and oligodendrocytes is still controversial. Elmore et al. (29) observed a slight increase in GFAP and S100 markers (at mRNA and protein level) (29, 37) but no changes in cell number or morphology (29, 38) after short-term treatment. However, Torres et al. (34) indicated that GFAP^+^ cells had thicker processes and higher intensity after seven days of treatment with PLX3397 (34). These results were consistent with Erblich et al. (39) that showed higher expression of GFAP and cell density in mice lacking CSF-1R (39).

Of interest, the action of drug-induced CSF-1R inhibition is reversible, meaning that withdrawal of the treatment allows the fast replenishment of the microglial population (40) from the resistant cells (13) without contribution from the bone marrow-derived cells. After near-complete depletion, repopulating microglia displayed enlarged cell bodies and a lack of ramifications within three days post-withdrawal (29, 40). After seven days, the microglia number increased by 160% of that in control mice, showing intermediate morphology and a cluster-like organization (13). By day 21 post-withdrawal, microglia returned to normal morphology and number (13, 40). Furthermore, Zhan et al. (41) indicated that PLX5622 withdrawal triggered the proliferation of the (Iba-1^+^) microglial cells and non-microglial (Iba-1^-^) cell populations within the first days of repopulation (13). They identified small subsets of Iba-1^-^ DCX^+^ and Iba-1^-^ Olig2^+^, markers of neurogenesis and oligodendrocytes, indicating that repopulation affects other resident cell types.

Comparison of gene expression indicated that BM-derived macrophages are highly different from steady-state microglia and, newly generated microglia after repopulation. Fewer differences were observed between control and newly repopulating microglial cells (32), indicating that repopulating microglia can keep most of their steady-state signature (13). However, morphologically, all three subtypes show similar cell body features. More detailed investigations revealed that repopulated microglial cells have a different transcriptome than resident microglia, showing upregulated cell-cycle (proliferation)-related gens Cdk1a and Mki67 and migration-related gene CD36 (32) but their impact on cell functionality has still to be investigated.

Additionally, Elmore et al. reported that control and newly repopulated microglia likely responded to lipopolysaccharide stimulation, indicating that both repopulated and steady-state microglia might also show similar reactivity and functional activity (42, 43). However, *ex vivo* analysis suggested that repopulated microglia showed reduced pro-inflammatory gene expression after stimulation to Toll-like receptor agonists (44), indicating that in some cases newly repopulated microglia might have attenuated pro-inflammatory activity, depending on the signalling molecules they sense.

Some depletion-repopulation paradigms in pathological conditions indicated that repopulation may resolve the activated microglial phenotypes and therefore, solve chronic microglia/macrophages-induced neuroinflammation (40, 45). Repopulation reduced almost half of the 46 genes overexpressed following neuronal lesion (40). Those genes were related to monocyte chemoattraction, endothelial transmigration of leukocytes and microglial proliferation, survival, phagocytic activity, and apoptotic pathway. It also resulted in the almost complete reversal of behavioural impairment observed with the elevated plus maze and Morris maze test. Similarly, (45) investigated the therapeutic effect of microglia depletion and repopulation during the chronic phase of experimental traumatic brain injury. They reported that short-term depletion followed by repopulation rescued microglia morphology, reduced neuroinflammation, oxidative stress, apoptosis and improved motor and cognitive functions (45).

On the other hand, there are also reports of absent therapeutic effects of microglia repopulation. In experimental autoimmune encephalomyelitis (EAE), drug withdrawal resulted in a rapid re-emergence of symptoms, leading eventually to peak scores comparable to those in control EAE mice, associated with an increase in microglia number 5-6 days after drug withdrawal (46). Moreover, the newly generated microglia triggered a degenerative inflammatory response upon their reappearance. Altogether, it seems that beneficial disease outcomes after CSF-1R inhibition-induced microglia repopulation are dependent on the disease model and therapy time window (46).

### The Colony Stimulating Factor-1 Receptor in Glioma

Immunotherapies represent a promising approach for treating cancer. Despite favorable results obtained treating different tumor types (47–50), they have not proven to be efficient in glioma so far. Treatment failure is likely related to the extensive spatial and temporal heterogeneity of the glioma microenvironment (51) and the numerous immunosuppressive mechanisms the tumor exploits, such as immune surveillance evasion.

Microglia are part of the innate immune response and are responsible for the phagocytosis of abnormal cells. However, in the tumour microenvironment (TME), they acquire a pro-tumorigenic phenotype under the influence of the tumour cells. Similarly, tumour-associated macrophages (TAMs) are differentiated from monocytes precursors recruited from the systemic reservoirs to the tumour in response to cytokines and chemoattractants released by tumour cells, including the CSF-1 ligand (52), ultimately supporting the immunosuppressive environment (53, 54). In glioma, these cells are also known as glioma-associated microglia/macrophages (GAMM) and represent around 30-50% of the total tumour mass (55). Single-cell profiling indicated that microglia and TAMs differentially contribute to the glioma environment over time, with an early microglial and late TAM contribution (51). GAMM favour tumour progression by releasing pro-tumorigenic, pro-survival and growth factors (56). They promote escape from the tumour immune response by boosting glioma angiogenesis, growth and invasion (57), suppression of cytotoxic T cell functions and induction of an immunosuppressive regulatory T (T_reg_) cell response (58). Additionally, GAMM has been associated with tumour progression and therapy resistance (59). Therefore, targeting GAMM may provide an important advantage over current standard therapy (60).

Efficient targeting of TAM using small molecule CSF-1R inhibitors was assessed in many tumour models, including solid tumours and breast cancers (61, 62). High levels of CSF-1 and CSF-1R have been observed in high-grade human glioma, supporting their pivotal role in tumour growth. The level of CSF-1 was correlated with tumorigenesis and increased GAMM density. Accordingly, targeting the CSF-1/CSF-1R axis may represent a potential therapeutic approach in glioma (63).

Significant reduction of TAM was achieved in different tumour models using CSF-1R inhibitors, partly due to the impaired recruitment and maturation of infiltrating monocytic TAMs precursors (64). While CSF-1R inhibition reduces the GAMM density, resistant cell populations were observed across different tumour types, including glioma (65, 66). Interestingly, Pyonteck et al. observed a substantial reduction in tumour growth, whereas Coniglio et al. reported a more subtle effect with decreased cell invasion and no effect on proliferation or survival, highlighting that CSF-1R inhibition therapeutic effects may depend on the glioma subtype (proneural vs mesenchymal). Nevertheless, the resistant TAM showed downregulated pro-tumorigenic markers expression, potentially slowing tumour progression in pancreatic cancer, cervical and mammary tumour and melanoma or improving the response to other treatments (67–69). In glioma models, CSF-1R inhibition delays recurrence and slightly prolonged overall survival (70) by altering the immune cell polarization state toward a less immunosuppressive phenotype (66). Different CSF-1R inhibitors and anti-CSF-1R antibodies have been tested in preclinical studies and clinical trials (71), to lower TAM burden, reprogram GAMM towards an anti-tumorigenic phenotype and stimulate the T-cells response (61). Despite promising results, they failed to show substantial efficacy across multiple tumor types (71) as well as in glioma (72, 73). Therapy resistance was predominantly associated with increased Foxp3^+^ Treg influx in response to macrophage depletion (74) and enhanced recruitment of other pro-tumorigenic cell populations such as myeloid-derived suppressor cells (75, 76). Those studies highlighted the main role of the TME in supporting therapy resistance.

**Figure 1:**
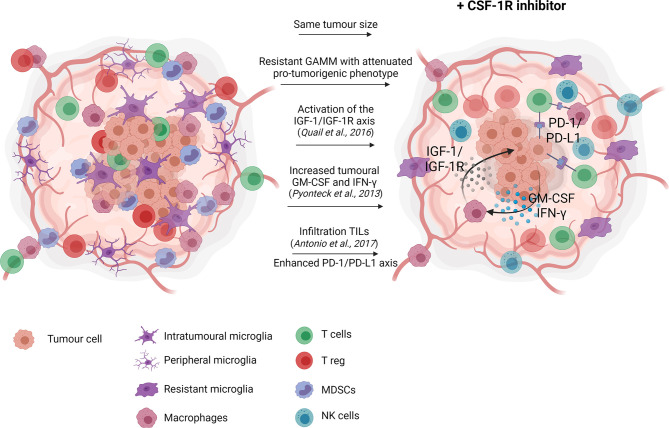
CSF-1R inhibition monotherapy in glioma. In glioma models, CSF-1R inhibition delays recurrence, and therefore slightly prolong overall survival with no significant effect on tumour growth. Reported resistance mechanisms to CSF-1R inhibition include increased insulin-like growth factor (IGF-1) signaling between macrophages and tumor cells, ultimately promoting tumor cell survival and invasion (77), increased levels of granulocyte-macrophage (GM)-CSF and interferon (IFN)- γ, leading to TAM persistence (66) and increased tumor-associated lymphocytes infiltration favoring the immunosuppressive PD-1/PD-L1 signaling (78). Created with Biorender.com.

CSF-1R monotherapy falls short in providing therapeutic effects due to acquired resistance (66, 76, 77) (**Figure 1**). In experimental glioma models, CSF-1R inhibition significantly prolonged overall survival while recurrence was observed in a considerable subset of animals. Acquired resistance to long-term CSF-1R inhibition was correlated with increased insulin-like growth factor (IGF-1) signaling between macrophages and tumor cells, leading to aberrant activation of phosphatidylinositol 3-kinase (PI3K) signaling, therefore promoting tumor cell survival and invasion (77). Glioma recurrence was also associated with increased levels of granulocyte-macrophage (GM)-CSF and interferon (IFN)- γ, leading to TAM persistence (66). Other studies reported that upregulation of the T cell immune checkpoint molecules, such as programmed cell death 1 ligand (PD-L1) and cytotoxic T-lymphocyte-associated protein 4 (CTLA-4), should also be considered as a potential escape mechanism from CSF-1R monotherapy (67, 78). Antonios et al. (78) demonstrated that CSF-1R therapy indirectly promotes tumor-infiltrating lymphocytes (TILs) recruitment within the glioma microenvironment (78). TILs are an important cellular source of PD-L1 expression and therefore, their infiltration could promote immune escape and resistance mediated by the PD-1/PD-L1 axis. Altogether, resistance to CSF-1R monotherapy may be explained by the cellular heterogeneity of the tumor microenvironment beyond GAMM. Accordingly, single-agent therapy with CSF-1R inhibitor has demonstrated very modest results in glioblastoma clinical trials, showing no significant improvement of the progression-free survival of the patients (79, 80).

Accordingly, ongoing studies are currently combining CSF-1R therapy and immune-checkpoint inhibitors in different types of tumors (81). CSF-1R therapy in cancer seems to have mostly a synergistic effect and improve other treatments, such as adoptive cell transfer immuno-therapy or platinum-based chemotherapy in breast cancer models (82, 83). In glioma, CSF-1R inhibition was combined with ionizing radiation and potentiated the response of the tumour to irradiation, indicated by decreased irradiation-induced monocytes recruitment, reduced pro-tumorigenic TAMs and longer survival (84). CSF-1R inhibitors are also reported to enhance the anti-tumoral T-cell responses when combined with immune-checkpoint inhibitors like anti-PD-1 antibodies (70, 78, 85). In glioma, the combination of both therapies increased cytotoxic CD8^+^/CD4^+^ and CD8^+^/FoxP3^+^ T cell ratios, indicative of an enhanced anti-tumour activity (70), leading to longer-term surviving animals. Interestingly, Ali and colleagues investigated different combinatorial therapies, considering CSF-1R, PD-1 and other targets, in glioblastoma and highlighted the importance of the therapy-induced time-dependent changes in TME cells (86). Therefore, further preclinical and clinical research should combine CSF-1R inhibition with other therapies to enhance therapeutic effects and investigate the optimal therapy paradigm.

### The Colony Stimulating Factor-1 Receptor in Stroke

Microglial cells play a significant role in initiating, maintaining, and resolving the inflammatory response after stroke. Microglia cells drastically change their morphology, gene expression, expression of inflammatory mediators, and surface molecule organization after detecting signs of injury such as intracellular calcium waves or ATP release. Based on the temporal changes of marker expression, microglial cells have potentially an early (beneficial) anti-inflammatory effect, while detrimental pro-inflammatory microglia seem to dominate at later stages (87). In experimental stroke, the level of anti-inflammatory markers peaked around day 4 post ischemia, while a wave of pro-inflammatory markers increased over the first two weeks, peaking around day 14 post ischemia (87).

**Figure 2:**
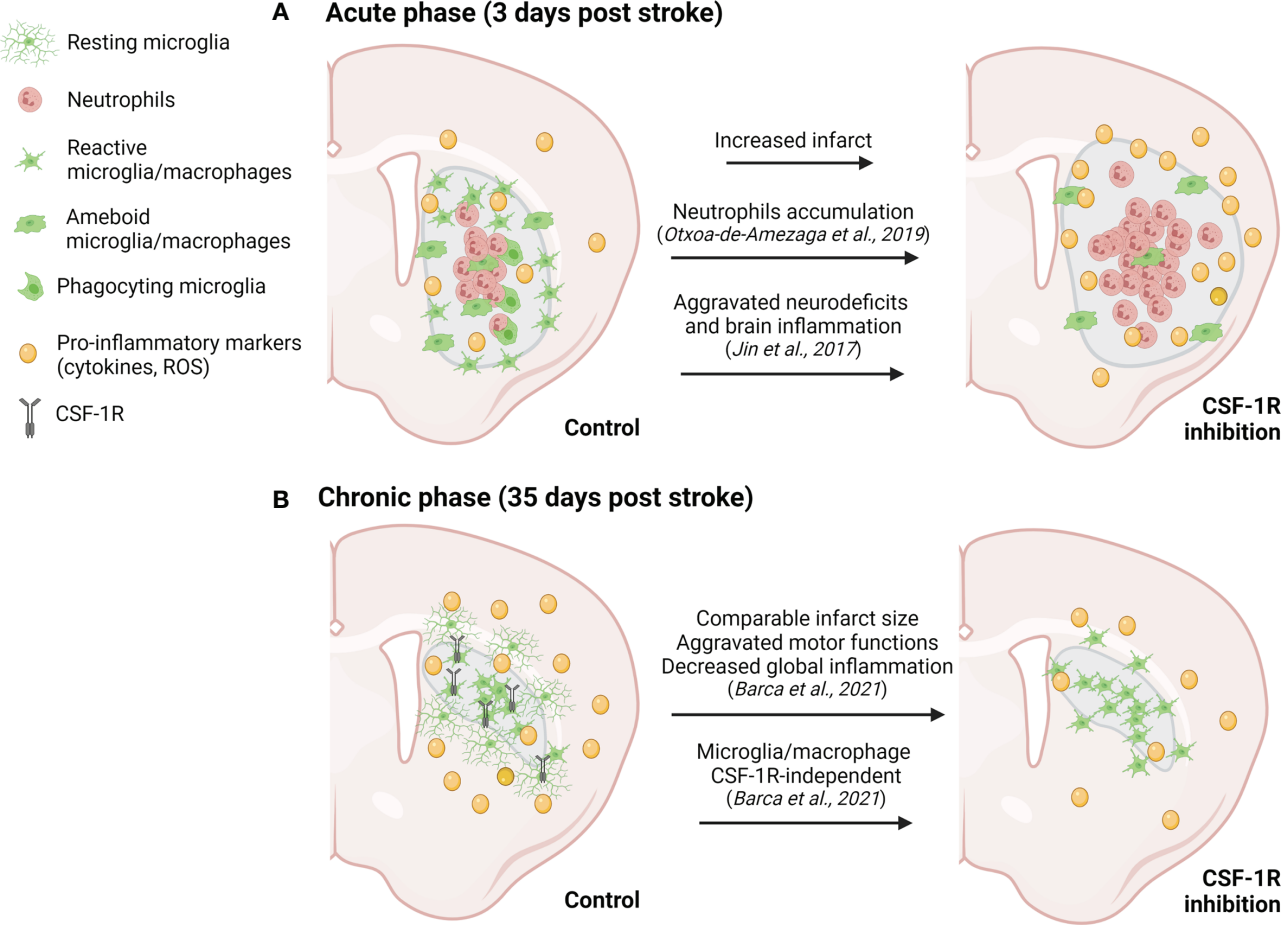
CSF-1R inhibition monotherapy in stroke. **(A)** Previous studies on brain pre-conditioning reported the absence of microglial cells within the first days post ischemia (acute phase) worsened disease outcomes, including increased brain injury, peripheral infiltration and pro-inflammatory signaling, ultimately leading to aggravated neurodeficits. **(B)** Long-term treatment reveals the existence of an Iba-1^+^ (microglia/macrophages) cell population resistant to CSF-1R inhibition while global expression of inflammation-related markers was decreased. Long-term CSF-1R inhibition starting right after surgery led to aggravated motor functions, partly explained by homeostatic imbalance and impaired infarct reperfusion (88). Created with Biorender.com.

PLX5622 and derivatives have been investigated in transient and permanent middle cerebral artery occlusion (MCAo) rodent models to understand how microglia depletion prior to stroke may affect disease outcomes. From the preventive studies, data confirmed that microglia might confer protection against injury at an early stage (**Figure 2A**). A 21-day PLX3397 microglia depletion prior to a transient middle cerebral artery occlusion (tMCAo) worsens disease outcomes, including increased brain injury, enhanced excitotoxicity and altered neuronal activity (89). Effect on brain injury/infarct size was also observed in a TBI mice model (90): depletion of microglia using PLX5622 (from 7 days before to 3 days after TBI) also increased the core size at day 3 (90).

In line with these findings, Wei Na Jin et al. (91) reported that uninterrupted PLX3397 treatment before and after MCAo exacerbated neurological deficits, brain inflammation (cytokine levels), cell death and leukocyte infiltration within the first days after ischemia (35, 91). Similarly, 35 reported continuous CSF-1R inhibition starting three weeks before ischemia increased the number of infiltrating neutrophils but reduced the numbers of monocytes (-40%), F4/80^+^ macrophages (-80%) at day 4 post ischemia (35). Additionally, increased CD4^+^T and NK cell counts correlated with a decrease of the corresponding leukocyte subsets in the spleen. Altogether, these studies suggest that the presence of CSF-1R^+^ cells has beneficial effects within the first 3 days post ischemia, reducing neurological deficits, cell death, ROS levels, leukocytes infiltration, neuroinflammatory markers (such as pro-inflammatory cytokines IL-1α, IL-1β, IL-6 and TNF-α) and increasing levels of growth factors (IGF-1) in some cases. Additionally, CSF-1R depletion prior to and after MCAo reduces the pro-inflammatory astrocytic reactivity (including IL-1α, IL-1β, iNOS, TNF-α, IL-6) with no change in astrocytes number (35, 91). Interestingly, Li et al., (11) used the same paradigm in an intracerebral haemorrhagic model (ICH model) and found the opposite results (11). Altogether, these studies support a neuroprotective role of microglial cells within the first days after stroke, which may partially be explained by its phagocytic and inflammatory activity on infiltrating cells at early stages.

Recently, our group assessed the immunomodulatory effect of long-term PLX5622 administration in the post ischemic phase using *in vivo* multimodal imaging (88). We demonstrated that CSF-1R inhibition transiently decreased neuroinflammation within the infarct, while a sustained decrease was observed in the contralateral healthy tissue, correlating with Iba-1^+^ (microglia/macrophages) dynamics. Interestingly, the decrease in activated microglia/macrophages number in remote areas such as the contralateral side may moderate the impact of spreading depression and ultimately global inflammation, as observed by the global decrease in pro- and anti-inflammatory markers expression in both hemispheres at late stage (**Figure 2B**). Moreover, long-term CSF-1R inhibition also affected homeostatic balance and tissue reperfusion, albeit transient, as indicated by diffusion- and perfusion-weighted MR imaging.

It is still unknown when and for how long microglia must be eliminated to enhance recovery: data may indicate that microglial activity is essential within the first days to reduce peripheral cell infiltration and cytotoxicity while it may become detrimental later on (87). Additionally, previous short-term CSF-1R inhibition studies in other disease models supported that PLX5622-induced microglia repopulation could reduce inflammatory cytokines expression, brain damage and resolve behavioural impairment. Altogether, those studies highlighted the importance of targeting microglia/macrophages within an optimal therapeutic time window to leverage their beneficial activity during the post ischemic phase. To date, no study on repopulation and/or short-term CSF-1R inhibition in stroke has been reported.

### *In Vivo* Molecular Imaging of CSF-1R

**Figure 3:**
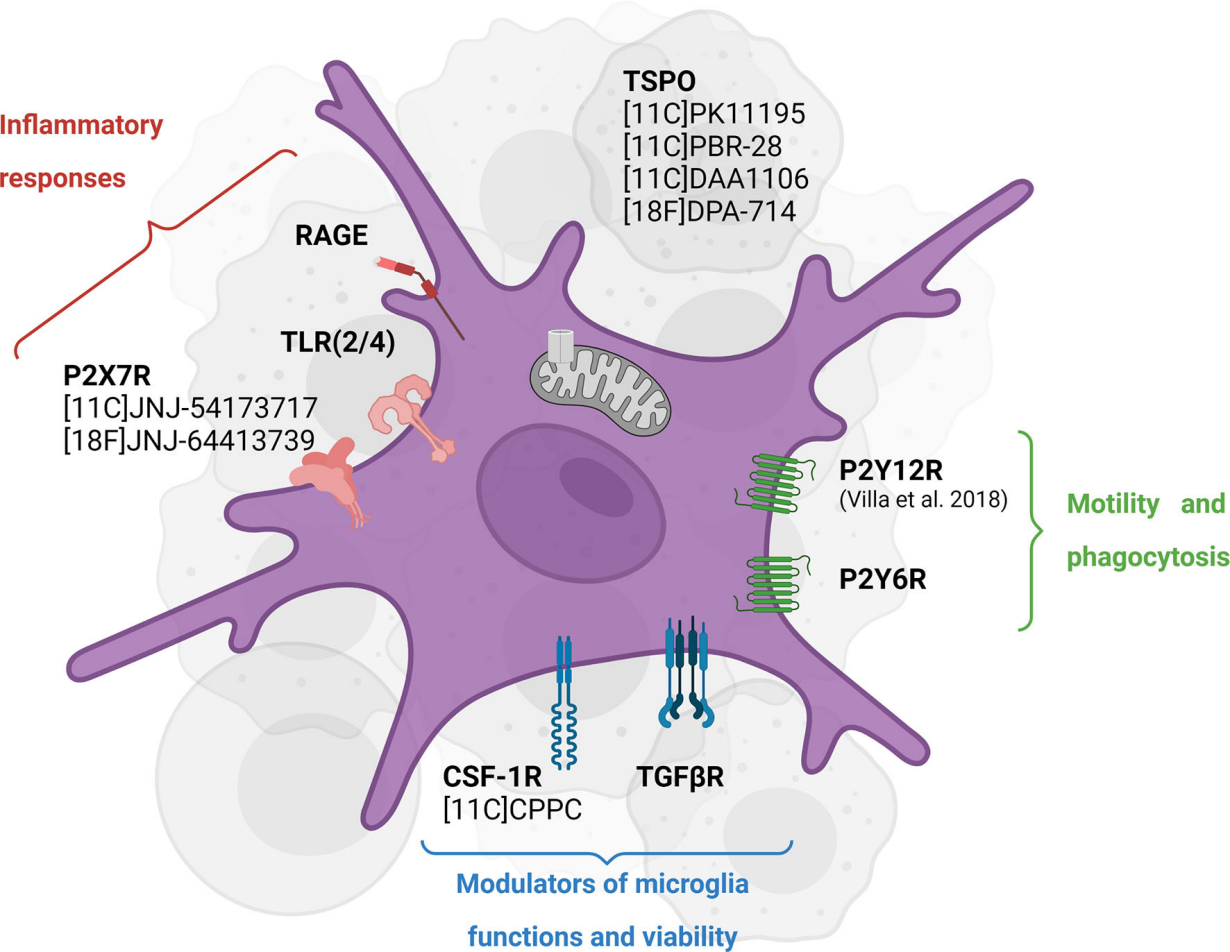
Emerging targets for *in vivo* imaging of CSF-1R inhibition-induced microglial activity modulation. TSPO PET tracers have been widely used to assess neuroinflammation in different pathologies while they have shown some caveats, including inability to distinguish cellular sources of TSPO and phenotypes. Some of the newly investigated targets including P2X7R and P2Y12R highlight the different functions of microglial cells in an inflammatory environment. Created with Biorender.com.

*In vivo* assessment of CSF-1R inhibition therapy response and target engagement would benefit from developing imaging probes specifically targeting CSF-1R and/or microglial cells (**Figure 3**).

In this context, Horti et al. (92) developed the new radiotracer 11C-CPPC targeting the CSF-1R. Preclinical assessments seem to indicate high selectivity and binding specificity in animal models of acute LPS-induced neuroinflammation and post-mortem Alzheimer’s disease human tissue (92) while others reported higher off-target binding and lower specificity (93). Therefore, other CSF-1R antagonist radioligands are currently investigated, potentially showing higher sensitivity and larger dynamic range in preclinical models (94). In addition, macrophage-targeted diagnostic tools are currently developed to visualize immune cell accumulation in a variety of inflammatory disease and assessed in the context of CSF-1R inhibition-induced microglia/macrophages depletion and other targeting immunotherapies (95).

The translocator protein (TSPO)-PET imaging has been widely used to study inflammation dynamics, immune cell activation and/or microgliosis in preclinical and clinical studies. Despite several well-known caveats, TSPO-PET imaging allows assessing global inflammation, visualizing areas of immune cell infiltration and defining tissue heterogeneity (96–98). Moreover, TSPO-PET has been used as a therapy readout in clinical trials in patients with primary glioblastoma or melanoma brain metastasis treated with chemoradiation or immunotherapy (NCT02431572). The validation of TSPO-PET tracers in clinical settings is necessary to improve the understanding of glioma-associated inflammation and microglia-targeting therapy resistance mechanisms. In a preclinical trial, seven days of CSF-1R inhibitor (PLX3397) in a non-human primate resulted in a significant reduction of 11C-PBR28 (TSPO) volumes of distribution by 46% from baseline, consistent with microglia depletion, which recovered after 12 days, supporting TSPO-PET as a CSF-1R inhibition therapy readout (99). However, the cellular sources of TSPO during or after treatment were not investigated. This finding encourages conducting back-translational studies to understand the biological mechanisms after CSF-1R therapy together with TSPO-PET imaging as a therapy readout.

Recently, our group reported the suitability of 18F-DPA-714 PET imaging, used as a biomarker of TSPO-dependent neuroinflammation and immune cell activation, to track the immunomodulatory effect of long-term PLX5622 administration in the post ischemic phase (88). We demonstrated that CSF-1R inhibition transiently decreased radiotracer uptake within the infarct, correlating with the dynamics of TSPO and microglia/macrophages *ex vivo*. Therefore, we supported the use of TSPO-PET imaging as a microglia-targeting therapy readout in stroke.

It should be noted that none of the TSPO radioligands is cell-specific or function-specific. Among the emerging targets, the purinergic metabotropic 12 receptor (P2Y12R) PET tracer is an attractive imaging biomarker to study microglial function (100, 101). P2Y12R expression is restricted to microglia in the CNS and absent on peripheral immune cells, involved in microglial chemotaxis and cytokine/chemokine signaling. In the inflammatory cascade, P2Y12R is downregulated in a pro-inflammatory environment and upregulated with exposure to anti-inflammatory stimuli, and therefore considered as a suitable biomarker for anti-inflammatory microglial cells (101). However, its temporal dynamics in an inflammatory environment remains not well understood (100): the specific role of P2Y12R seems to be disease- and stage-dependent. To our knowledge, only one P2Y12R-PET radioligand has been developed, the ethyl6-(3-(3-((5-chlorothiophen-2-yl)sulfonyl)11C-ureido)azetidin-1-yl)-5-cyano-2-methylnicotinate (101). While preliminary data looked promising, this tracer revealed low metabolic stability and lack of blood-brain barrier permeability. Further preclinical studies must assess other CNS-penetrant P2Y12R receptor PET radioligand and investigate the functional spatiotemporal role of P2Y12R.

As part of the same receptor family, the purinergic P2X7 receptor is found to be specifically upregulated in pro-inflammatory activated microglial cells in response to high ATP release. This receptor mediates cytokine and chemokines release, regulates T lymphocytes survival and differentiation (Di 102). A clinical trial investigated the blocking effect of JNJ-55308942 targeting the P2X7 receptor using the 18F-labelled analog in healthy volunteers (NCT03437590). The preliminary results in humans supported the use of the PET-tracer 18F-JNJ-54175446 to provide an insight into P2X7R in health and disease (103). In the context of CSF-1R inhibition, further studies may consider using this radioligand to assess (i) the spatiotemporal expression of P2X7 receptor in pathological conditions, (ii) the decrease in P2X7-expressing pro-inflammatory microglial cells following CSF-1R- and/or any microglia-targeting immunotherapy and (iii) the potential anti-inflammatory effect of microglia repopulation.

## Conclusion Remarks

Glioma and stroke are two complex pathological conditions, both inducing strong and chronic inflammatory and immunological responses following alterations of the immune balance. However, innate, and adaptive immune response differs in major aspects: origin, progression, and disease-induced phenotype. The comparison of both diseases represents a very interesting avenue of research as they represent the two extremes of neurological conditions. Stroke on the one hand with a strong pro-inflammatory stimulus (hypoxia, reperfusion) and on the other hand gliomas with a strong anti-inflammatory immunosuppressive microenvironment. The comparison thus supports evidence generation for the role of CSF-1R across neurological diseases.

CSF-1R inhibition studies demonstrate that many discernible subpopulations of microglial cells co-exist in the inflammatory environment and are differentially sensitive to CSF-1R inhibition. In both glioma and ischemia, a resistant population of CSF-1R-independent microglia/macrophages were observed after long-term PLX5622 treatment. Their contribution to compensatory mechanisms or therapy resistance still needs further research. One line of investigation focuses on the enhanced communication with other immune players, triggering modulation of the peripheral immune cell infiltration or activation of resident cells. Still, numerous compensatory and resistance mechanisms seem to be implemented by immunological responses beyond microglia/macrophages cells, limiting the efficiency of CSF-1R inhibition as a monotherapy. Therefore, research supports its use as a combination therapy to synergize the therapeutic effects of other immunomodulatory approaches.

Recently, we validated the use of TSPO-PET in preclinical studies assessing the therapeutic effect of new microglia-targeting treatments. TSPO-PET employing 18F-DPA-714 allows visualization of microglia/macrophages depletion-repopulation and areas of immune cell infiltration. However, a better knowledge of the therapeutic effects on other immune cell populations after short-term or long-term CSF-1R inhibition would improve our understanding of the *in vivo* TSPO dynamics upon therapy. Advances in microglia-targeting immunotherapy should boost the development of microglia-specific PET radioligands. This could support further clinical trials for glioma and stroke patients, improve personalized management and understand the prognostic value of multimodal imaging in microglia-targeting therapeutic approaches.

## Author Contributions

All authors conceptualized and wrote this manuscript. All authors contributed to the article and approved the submitted version.

## Funding

This work was partly funded by the Horizon 2020 Programme under grant agreement n°675417 (PET3D) and the Herbert-Worch-Stiftung. Additionally, this work was supported by a Collaboration Grant of the Medical Faculty of the University of Bonn between CIO UKB and Johanniter Hospital. This review was supported by the Immune-Image consortium. The Immune-Image project receives funding from the Innovative Medicines Initiative 2 Joint Undertaking (JU) under grant agreement No 831514 (Immune-Image). The JU receives support from the European Union’s Horizon 2020 research and innovation programme and EFPIA.

## Conflict of Interest

The author BZ is currently employed by F. Hoffman-La Roche Ltd.

The remaining authors declare that the research was conducted in the absence of any commercial or financial relationships that could be construed as a potential conflict of interest.

## Publisher’s Note

All claims expressed in this article are solely those of the authors and do not necessarily represent those of their affiliated organizations, or those of the publisher, the editors and the reviewers. Any product that may be evaluated in this article, or claim that may be made by its manufacturer, is not guaranteed or endorsed by the publisher.
